# The independent, unfavorable prognostic factors endothelin A receptor and chemokine receptor 4 have a close relationship in promoting the motility of nasopharyngeal carcinoma cells via the activation of AKT and MAPK pathways

**DOI:** 10.1186/1479-5876-11-203

**Published:** 2013-08-29

**Authors:** Dong-Hua Luo, Qiu-Yan Chen, Huai Liu, Li-Hua Xu, Hui-Zhong Zhang, Lu Zhang, Lin-Quan Tang, Hao-Yuan Mo, Pei-Yu Huang, Xiang Guo, Hai-Qiang Mai

**Affiliations:** 1 State Key Laboratory of Oncology in South China, Guangzhou, Guangdong 510060, P. R. China; 2 Department of Nasopharyngeal Carcinoma, Sun Yat-sen University Cancer Center, 651 Dongfeng Road East, Guangzhou, Guangdong 510060, P. R. China; 3 Department of Pathology, Sun Yat-sen University Cancer Center, Guangzhou, Guangdong 510060, P. R. China; 4 Center of Oncology and Hematology, The First Affiliated Hospital of Guangzhou Medical College, Guangzhou P. R. China

**Keywords:** Nasopharyngeal carcinoma, Prognosis, ETAR, CXCR4, Metastasis

## Abstract

**Background:**

Recent studies have indicated that the expression of endothelin A receptor (ETAR) and chemokine receptor 4 (CXCR4) could be used as an indicator of the metastatic potential of nasopharyngeal carcinoma (NPC). The aim of this study was to determine the prognostic value of ETAR and CXCR4 in NPC patients and to reveal the interplay of the endothelin-1 (ET-1)/ETAR and stromal-derived factor-1(SDF-1)/CXCR4 pathways in promoting NPC cell motility.

**Methods:**

Survival analysis was used to analyze the prognostic value of ETAR and CXCR4 expression in 153 cases of NPC. Chemotaxis assays were used to evaluate alterations in the migration ability of non-metastatic 6-10B and metastatic 5-8F NPC cells. Real-time PCR, immunoblotting, and flow cytometric analyses were used to evaluate changes in the expression levels of CXCR4 mRNA and protein induced by ET-1.

**Results:**

The expression levels of ETAR and CXCR4 were closely related to each other and both correlated with a poor prognosis. A multivariate analysis showed that the expression levels of both ETAR and CXCR4 were independent prognostic factors for overall survival (OS), progression-free survival (PFS), and distant metastasis-free survival (DMFS). The migration of 6-10B and 5-8F cells was elevated by ET-1 in combination with SDF-1α. The knockdown of ETAR protein expression by siRNA reduced CXCR4 protein expression in addition to ETAR protein expression, leading to a decrease in the metastatic potential of the 5-8F cells. ET-1 induced CXCR4 mRNA and protein expression in the 6-10B NPC cells in a time- and concentration-dependent fashion and was inhibited by an ETAR antagonist and PI3K/AKT/mTOR and MAPK/ERK1/2 pathway inhibitors.

**Conclusions:**

ETAR and CXCR4 expression levels are potential prognostic biomarkers in NPC patients. ETAR activation partially promoted NPC cell migration via a mechanism that enhanced functional CXCR4 expression.

## Background

Nasopharyngeal carcinoma (NPC) is most prevalent in southern China and Southeast Asia, regions where the incidence rate of NPC is 25–50 per 100,000 people [1-3]; by comparison, the incidence is less than 1 per 100,000 in North America and other Western countries [2]. NPC is notorious for its potential to metastasize via both lymph and blood vessels during the early stages of the disease [4]. Although the cervical lymph nodes are the primary sites of NPC metastasis [5], a considerable proportion of patients will develop distant metastases to the bone, lung, and liver [6], and distant metastasis after treatment is the major cause of treatment failure [7]. Moreover, the mechanisms that control NPC metastasis remain poorly understood.

Recent studies have revealed that the endothelin-1 (ET-1)/endothelin A receptor (ETAR) axis is related to the prognosis of cancer patients. Indeed, the serum ET-1 level was correlated with distant metastasis in NPC patients [8], and the ETAR inhibitor ABT-627 was found to inhibit the experimental metastasis of NPC cells [9]. The engagement of ETAR by ET-1 triggers the activation of tumor proliferation [10-14], vascular endothelial growth factor (VEGF)-induced angiogenesis [15,16], invasiveness [17], and the inhibition of apoptosis [18,19]. The autocrine ET-1/ETAR pathway has a key role in the development and progression of prostate [10], cervical [12], and ovarian [13] cancers. These findings support a role for the ETAR pathway in tumorigenesis and tumor progression. Furthermore, data from *in vitro* and *in vivo* studies have demonstrated that ETAR is a potential antitumor target [12].

The metastasis of cancer cells is a complex, highly organized, non-random, and organ-selective process. A complex network of chemokines and their receptors influence the development of primary tumors and metastases [20-23]. Recent studies have clearly demonstrated the importance of chemokine receptor (CR) expression in metastasis to specific organs (e.g., lymph nodes, bone marrow, liver, and lungs) by breast cancer [23], melanoma [24], and gastric carcinoma [25] cells. SDF-1 (CXCL12) and its receptor, chemokine receptor 4 (CXCR4), play an important role in tumor cell proliferation, migration, adhesion, extracellular matrix degradation, angiogenesis, and immune tolerance induction [26], and CXCR4 expression is associated with a poor overall survival (OS) in NPC patients [27]. Additionally, the expression of functional CXCR4 is associated with the metastatic potential of human NPC cells [28].

Both ETAR and CXCR4 expression can affect the metastatic capability of NPC cells. However, the relationship between ETAR and CXCR4 expression remains unclear, and the interplay of the ET-1/ETAR and SDF-1/CXCR4 pathways is unknown. A report by Masumi Akimoto et al. [29] showed that the expression levels of CXCR4 and ETAR are both increased in the healing and scarring stages of gastric ulcers, and these receptors have therefore been suggested to play a role in vascular maturation and gastric mucosal regeneration during late angiogenesis. In the present study, we investigated the relationship between ETAR and CXCR4 expression in NPC tissue and an NPC cell line. We found that ETAR and CXCR4 were closely related to each other and were related to the development of distant metastasis and a poor patient prognosis. We further investigated whether ETAR activation could increase functional CXCR4 expression in human NPC cells (6-10B and 5-8F). Our experimental study showed that ET-1 promotes the expression of functional CXCR4 in non-metastatic human NPC 6-10B cells and metastatic 5-8F cells and increases the migration ability of these cells through the PI3K/AKT and MAPK/ERK1/2 pathways.

## Patients and methods

### Patients

Between February 1999 and October 2000, 153 consecutive patients with non-metastatic NPC, who were hospitalized in the Department of NPC, Sun Yat-sen University Cancer Center, were enrolled in this study. All patients had biopsy-proven World Health Organization (WHO) type III NPC, which is an undifferentiated, non-keratinizing carcinoma. The study was approved by the Clinical Research Ethics Committee of the Sun Yat-sen University Cancer Center, and written informed consent was obtained from all patients. The AJCC 1997 staging system was used for clinical staging. All the recruited patients were treated with a uniform radiotherapy protocol, as described previously [30]. After completion of the treatment, the patients were followed up at least every 3 months during the first 3 years and then every 6 months thereafter until death. The patient follow-up was performed until February 2012. The median duration of follow-up for the entire group was 83.3 months (range of 3.4-148 months). The patients and clinicopathological characteristics are described in Table 1.

**Table 1:** Correlation of ETAR/CXCR4 expression with prognostic factors in patients with nasopharyngeal carcinoma

	**Entire group**	**CXCR4 expression**			**ETAR expression**		
	**(n = 153)**	**n (No.%)**			**n (No.%)**		
**Characteristic**		**Positive**	**Negative**	***P *value**	**Positive**	**Negative**	***P *value**
**All patients**	**153**	**48(31.4)**	**105(68.6)**		**113(73.9)**	**40(26.1)**	
**Mean follow-up, months**	**83.27**	**62.77**	**92.64**		**79.81**	**93.05**	
**Age, years**				**0.049**			**0.741**
**≤50**	**100(65.4)**	**26(26.0)**	**74(74.0)**		**73(73.0)**	**27(27.0)**	
**>50**	**53(34.6)**	**22(41.6)**	**31(58.4)**		**40(75.5)**	**13(24.5)**	
**Sex**				**0.27**			**0.213**
**Male**	**111(72.5)**	**32(28.8)**	**79(71.2)**		**85(76.6)**	**26(23.4)**	
**Female**	**42(27.5)**	**16(38.1)**	**26(61.9)**		**28(66.7)**	**14(33.3)**	
**T classification**^a^				**0.098**			**0.517**
**T1-2**	**66(43.1)**	**16(24.2)**	**50(75.8)**		**47(71.2)**	**19(28.8)**	
**T3-4**	**87(56.9)**	**32(36.8)**	**55(63.2)**		**66(75.9)**	**21(24.1)**	
**N classification**^a^				**0.681**			**0.284**
**N0**	**32(20.9)**	**11(34.4)**	**21(65.6)**		**26(81.3)**	**6(19.7)**	
**N1-N3**	**121(79.1)**	**37(30.6)**	**84(69.4)**		**87(71.9)**	**34(29.1)**	
**Overall stage**^a^				**0.376**			**0.94**
**I-II**	**49(32.0)**	**13(26.5)**	**36(73.5)**		**36(73.5)**	**13(26.5)**	
**III-IV**	**104(68.0)**	**35(33.7)**	**69(66.3)**		**77(74.0)**	**27(26.0)**	
**Chemotherapy**				**0.394**			**0.362**
**Yes**	**47(30.7)**	**17(36.2)**	**30(63.8)**		**37(78.7)**	**10(21.3)**	
**No**	**106(69.3)**	**31(29.2)**	**75(70.8)**		**76(71.7)**	**30(28.3)**	

Note: All patients were classified with World Health Organization (WHO) type III NPC.

^a^UICC 1997 staging system.

### Immunohistochemical analysis

Tumor specimens from the 153 patients were obtained by a pretreatment nasopharyngeal biopsy. The specimens were fixed in 10% formalin and embedded in paraffin, and immunohistochemical staining of these samples was performed as previously described [8,9]. Briefly, 4-μm-thick tissue sections were deparaffinized with xylene and rehydrated in a graded series of ethanol. The endogenous peroxidase activity was blocked with 3% hydrogen peroxide, and the sections were then subjected to antigen retrieval in a microwave oven using a citrate buffer solution. After blocking with normal goat serum for 10 minutes, the samples were incubated with a polyclonal rabbit anti-ETAR antibody (1:100 dilution; Santa Cruz) or a monoclonal mouse anti-CXCR4 antibody (MAB 172; dilution 1:600; R&D Systems) at 4°C overnight. The sections were then incubated with a biotin-labeled secondary antibody and streptavidin-peroxidase for 30 minutes each (Zhongshan Biotechnology, Beijing, China). Antibody binding was visualized using a freshly prepared solution of 0.04% 3′, 3′-diaminobenzidine tetrahydrochloride and 0.03% hydrogen peroxide and then counterstained with hematoxylin; the samples were then cleaned and mounted. The negative controls were stained similarly, except that serum from a non-immunized rabbit was used in place of the primary antibodies. Specimens of prostate cancer with ETAR-positive cancer tissue were used as a positive control.

The ETAR immunoreactivity was evaluated according to the percentage of stained cancer cells and the staining intensity, which was classified into the following two groups: positive, with more than 50% of tumor cells having intense cytoplasmic staining, and negative, representing other patterns of lower staining [31]. The expression of ETAR was characterized as negative (−) or positive (+) by one of the authors (H. Z. Zhang), who had no prior knowledge of any of the clinical or radiological data.

CXCR4 positivity was graded semi-quantitatively according to Carcangiu’s method [32] as weak or absent (total score ≤3) or strong (total score ≥4) by one of the authors (H. Z. Zhang), without prior knowledge of the clinicopathological features or the clinical follow-up data of the patients.

### Cell culture

Non-metastatic human 6-10B cells and metastatic 5-8F cells [33] were obtained from the Department of Experimental Research, Sun Yat-sen University Cancer Center. The cells were cultured in RPMI 1640 medium supplemented with 1% penicillin/streptomycin (Invitrogen) and 10% FBS. All of the cells were maintained in 10-cm tissue culture dishes in a 37°C incubator equilibrated with 5% CO_**2**_ in humidified air.

### Flow cytometry

Initially, the 6-10B cells were serum-starved for 24 hours and then stimulated with increasing concentrations (0, 0.1, 1, 10, and 100 nM) of ET-1 (Sigma) for 24 hours or with 10 nM ET-1 for the time indicated. The cells were then grown to subconfluence, detached with cold Dulbecco’s PBS (5 mmol/L EDTA), and washed with fluorescence-activated cell-sorting buffer (5 mmol/L EDTA, 0.1% NaN3, and 1% FCS in Dulbecco’s PBS). After incubation with a monoclonal antibody against human CXCR4 (R&D Systems) for 30 minutes on ice, the cells were stained with an FITC-labeled secondary antibody and examined for CXCR4 expression using flow cytometry (BD Biosciences).

### Western blotting

Cell lysates from selected 6-10B and 5-8F clones were prepared using standard procedures. The concentration of total protein was determined using a BCA assay (Pierce). Loading buffer was added to the protein (40 μg) samples, which were boiled prior to resolution by SDS-PAGE on 12% gels; the proteins were then transferred onto PVDF membranes (Bio-Rad). The blots were blocked for 2 hours with blocking reagent while shaking and then incubated with a primary antibody against CXCR4 (1:1000 dilution; Biolegend), ERK (1:1000 dilution; Santa Cruz), P-ERK (1:1000 dilution; Cell Signaling), AKT (1:1000 dilution; Santa Cruz), P-AKT (1:1000 dilution; Santa Cruz), alpha tubulin (1:1000 dilution; Cell Signaling), or GAPDH (1:1500 dilution; PTG). The blots were washed and incubated for 2 hours with the corresponding secondary antibodies (Dako). A rabbit anti-mouse antibody was used at 1:6000 for CXCR4, and a swine anti-rabbit antibody was used at 1:6000 for ERK, P-ERK, AKT, P-AKT, and GAPDH. After washing, the immunoreactive bands were visualized with Super Signal West Dura Extended Duration Substrate Enhanced Chemiluminescent Substrate (Pierce Biotechnology). Each assay was performed independently and in triplicate. As a control for equal protein loading, immunoblotting for GAPDH or alpha tubulin were performed on the membranes after stripping the previous antibodies. The levels of CXCR4, ERK, P-ERK, AKT, and P-AKT were normalized to that of GAPDH.

### Real-time PCR

Prior to the PCR analysis, 6-10B cells were serum-starved for 24 hours and then stimulated with increasing concentrations (0, 0.1, 1, 10, and 100 nM) of ET-1 (Sigma) for 24 hours or with 10 nM ET-1 for the time indicated. Total RNA was extracted from selected 6-10B clones using TRIzol reagent (Life Technologies); a genomic DNA removal kit (RNeasy Plus Mini Kit, Qiagen GmbH) was used to remove any DNA from the sample. The total RNA was then subjected to real-time RT-PCR using an iCycler iQ Multicolor Real-Time PCR Detection System (Bio-Rad) with the iScript one-step RT-PCR kit with SYBR Green (Bio-Rad). A melting curve analysis was performed to evaluate the purity of the PCR products; triplicate samples were evaluated for each primer set. The expression of CXCR4 relative to GAPDH (a housekeeping control gene) was calculated using the ΔCT method. The following CXCR4 primers were utilized: sense, 5′-CCAACGTCAGTGAGGCAGAT-3′, and antisense, 5′-GGCAGGATAAGGCCAACCAT-3′. The following GAPDH primers were used: sense, 5′-AGCCTCAAGATCATCAGC-3′, and antisense, 5′-GAGTCCTTCCACGATACC-3′.

### siRNA and transfections

The following siRNAs were purchased from Santa Cruz Biotechnology, Inc.: siETAR (ETAR siRNA (h): sc-39960) and siCXCR4 (CXCR-4 siRNA (h): sc-35421). The siRNA transfection protocol is available online at http://datasheets.scbt.com/siRNA_protocol.pdf.

### Chemotaxis assays

Chemotaxis assays were performed using 48-well chemotaxis chambers (Corning, USA). Aliquots of 27 to 29 μL of assay medium (RPMI 1640 containing 1% bovine serum albumin and 30 mmol/L HEPES) with 100 nM SDF-1α (Merck) were placed in the lower wells of the chamber, and a 200-μL cell suspension (5 × 10^4^ cells/mL) aliquot was placed in the upper wells. The 6-10B cells were serum-starved and then stimulated with increasing concentrations (0, 0.1, 1, 10, and 50 nM; Sigma) of ET-1 for 12 hours with SDF-1α (100 nM) in the lower chamber of the assay. ETAR or CXCR4 expression was knocked down in the 5-8F cells, which were then stimulated or not with ET-1 (10 nM). The upper and lower wells were separated using a polycarbonate filter (10-μm pore size; Osmonics), which was pre-coated with 50 μg/mL collagen type I (Collaborative Biomedical Products). After incubation at 37°C for 12 hours, the filter was removed and stained, and the cells that had migrated across the filter were counted under a light microscope after coding the samples. The results were expressed as the chemotaxis index, which represents the fold increase in the number of migrated cells in response to chemoattractants over spontaneous cell migration in response to the control medium.

### Statistical analysis

SPSS 13.0 was used for the statistical analysis. Survival was calculated using the Kaplan-Meier method, and the resulting curves were compared using the log-rank test. Fisher’s exact test and the chi-square test were used to analyze the association between two categorical variables. The Cox proportional hazard model was used to perform a multivariate analysis of the risk factors for patient prognosis. *P* < 0.05 was considered to be statistically significant.

All the experiments were performed at least three times, and representative results are shown. The significance of the differences between various groups was analyzed with Student’s t test or the chi-square test.

## Results

### The positive correlation between ETAR and CXCR4 expression in NPC tissue samples

**Figure 1:**
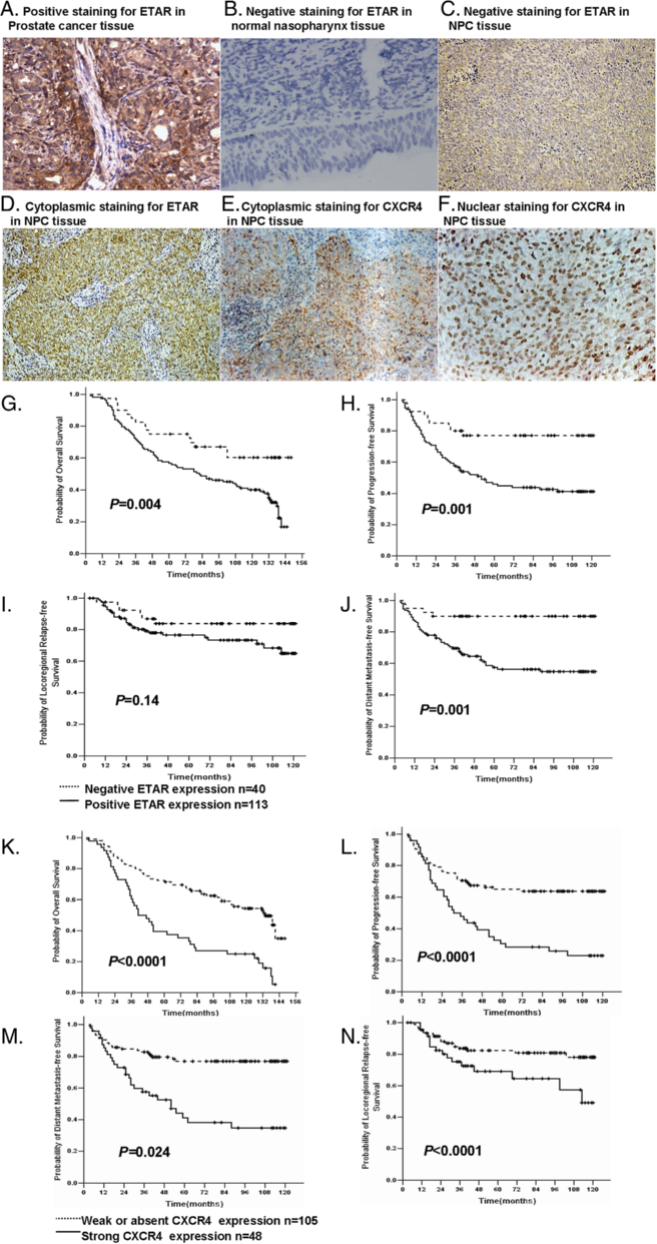
**ETAR/CXCR4 expression levels and their prognostic value in undifferentiated NPC. A-F**. Immunohistochemical staining pattern of ETAR/CXCR4 expression. **A**. Positive staining for ETAR in prostate cancer tissue. **B**. Negative staining for ETAR in normal nasopharynx tissue. **C**. A case of negative staining for ETAR. **D**. A case showing strong (≥50%) cytoplasmic staining for ETAR. **E**. A case showing strong cytoplasmic staining for CXCR4. **F**. A case showing strong nuclear staining for CXCR4. Original magnification, × 200. **G**-**N**. Kaplan-Meier estimates of survival curves based on the ETAR/CXCR4 expression levels of all 153 patients. **G-J**. Based on the ETAR expression levels. **G**. Overall survival, *P* = 0.004. **H**. Progression-free survival, *P* = 0.001. **I**. Locoregional-relapse-free survival, *P* = 0.14. **J**. Distant-metastasis-free survival, *P* = 0.001. **K-N**. Based on the CXCR4 expression levels. K, Overall survival, *P* < 0.0001. L, Progression-free survival, *P* < 0.0001. **M**. Locoregional-relapse-free survival, *P* = 0.024. **N**. Distant-metastasis-free survival, *P* < 0.0001.

Using prostate cancer tissue as a positive control (Figure 1A) [31], ETAR expression was present in 73.9% (113/153) of the tumor samples, whereas 14 cases (14/18, 77.8%) of normal nasopharyngeal tissues were negative for ETAR expression (Figure 1B). The intensity of staining was variable among the samples, ranging from absent or weak to strong (Figure 1C, D), and the ETAR immunoreactivity was mainly detected in the cytoplasm of the carcinoma cells (Figure 1D). Strong CXCR4 expression was detected in 31.4% (48/153) of the cancer samples (Figure 1E, F), whereas the remaining 105 samples displayed weak or absent CXCR4 staining. The ETAR and CXCR4 expression levels were closely correlated with each other: in the 48 NPC cases positive for the expression of CXCR4, 46 (95.8%, *P* < 0.0001) were also positive for ETAR expression (R = 0.338, *P* = 0.0001) (Table 2).

**Table 2:** Correlation between CXCR4 and ETAR immunohistochemical expression

		**ETAR**		
**CXCR4**	**No**	**-**	**+**	***P *value**
**-**	**105**	**38**	**67**	**<0.0001**
**+**	**48**	**2**	**46**	**<0.0001**
**Total**	**153**	**40**	**113**	

### The correlation between ETAR and CXCR4 and their prognostic value

The 5-year OS, progression-free survival (PFS), locoregional relapse-free survival (LRRFS), and DMFS rates in the ETAR-positive patients were 56.6%, 45.9%, 76.5%, and 57.4%, respectively. The corresponding rates in the ETAR-negative patients were 75.0%, 77%, 83.7%, and 90%, respectively. With the exception of locoregional failure, all the differences were statistically significant (Figure 1I). No correlation was found between ETAR expression and the gender, age, T stage, N stage, or TNM clinical stage of the patients (*P* > 0.05; Table 1).

Next, we analyzed the relationship between the clinical outcome and CXCR4 expression levels. The 5-year OS, PFS, LRRFS, and DMFS rates in the CXCR4-positive patients were 39.6%, 30.6%, 69.1%, and 41.1%, respectively; the corresponding rates were 71.4%, 64.9%, 82.4%, and 76.9%, respectively, in the CXCR4-negative patients. All the differences were statistically significant (Figure 1K-N). No correlation was found between the CXCR4 expression levels and gender, age, N stage, or TNM clinical stage of the patients (*P* > 0.05; Table 1). However, CXCR4 expression did show a positive correlation with T stage (R = 0.1688, *P* = 0.038).

To adjust for prognostic factors, the following parameters were included in the multivariate analysis using the Cox proportional hazards model: gender (male vs. female), age (≤50 vs. >50 years), T stage (T1-2 vs. T3-4), N stage (N0-1 vs. N2-3), clinical stage (stage I-II vs. stage III-IV), ETAR expression (negative vs. positive), and CXCR4 expression (weak/absent vs. strong ). A stepwise forward procedure was used for the analyses. By including the ETAR and CXCR4 expression levels separately in the Cox model, along with other variables, the multivariate analysis showed that the expression of ETAR was an independent prognostic factor for OS (HR, 1.93; 95% CI, 1.10-3.38; *P* = 0.02), PFS (HR, 2.58; 95% CI, 1.28-5.22; *P* = 0.008), and DMFS (HR, 4.62; 95% CI, 1.66-2.86; *P* = 0.003) (Table 3) and that the expression of CXCR4 was an independent significant prognostic factor for OS (HR, 2.78; 95% CI, 1.78-4.35; *P* < 0.001), PFS (HR, 2.67; 95% CI, 1.67-4.28; *P* < 0.001), and DMFS (HR, 3.35; 95% CI, 1.90-5.92; *P* < 0.001) (Table 4). When ETAR and CXCR4 were included together in the Cox model, along with other variables, the results showed that CXCR4 expression was an independent prognostic factor for OS (HR, 2.78; 95% CI, 1.78-4.35; *P* < 0.001) and that both ETAR (HR, 2.12; 95% CI, 1.03-4.36; *P* = 0.04) and CXCR4 (HR, 2.30; 95% CI, 1.42-3.72; *P* < 0.001) expression were independent prognostic factors for PFS. For DMFS, N stage (HR, 1.96; 95% CI, 1.48-2.59; *P* < 0.001), ETAR expression (HR, 3.46; 95% CI, 1.22-9.84; *P* = 0.02), and CXCR4 expression (HR, 2.52; 95% CI, 1.42-4.46; *P* = 0.002) were independent prognostic factors. In contrast, clinical stage was the only independent, significant prognostic factor for LRRFS (HR, 1.76; 95% CI, 1.22-2.54; *P* = 0.002) (Table 5).

**Table 3:** Summary of the multivariate analysis of prognostic factors in nasopharyngeal carcinoma (Excluding CXCR4)

**Clinical endpoint**	**Prognosticator**	**HR**	**95% CI**	***P *value**
**OS**	ETAR expression	1.93	1.11-3.38	0.02
	UICC stage	1.54	1.19-2.01	0.001
	N classification	1.42	1.12-1.80	0.003
	Gender	0.57	0.34-0.93	0.026
	Age	1.98	1.28-3.07	0.002
**PFS**	ETAR expression	2.58	1.28-5.22	0.008
	UICC stage	1.36	1.02-1.84	0.04
	N classification	1.42	1.1-1.84	0.007
	Gender	0.47	0.25-0.89	0.02
**DMFS**	ETAR expression	4.62	1.66-12.86	0.003
	N classification	1.92	1.46-2.54	<0.001
**LRRFS**	UICC stage	1.76	1.22-2.54	0.002

*CI* confidence interval, *DMFS* distant metastasis-free survival, *HR* hazard ratio, *LRRFS* locoregional relapse-free survival, *OS* overall survival, *PFS* progression-free survival.

**Table 4:** Summary of the multivariate analysis of prognostic factors in nasopharyngeal carcinoma (Excluding ETAR)

**Clinical endpoint**	**Prognosticator**	**HR**	**95% CI**	***P *value**
**OS**	CXCR4 expression	2.78	1.78-4.35	<0.001
	UICC stage	1.51	1.15-1.98	0.003
	N classification	1.47	1.16-1.88	0.002
	Gender	0.48	0.29-0.80	0.004
	Age	1.65	1.07-2.56	0.02
**PFS**	CXCR4 expression	2.67	1.67-4.28	<0.001
	N classification	1.64	1.3-2.07	<0.001
	Gender	0.36	0.20-0.69	0.00
**DMFS**	CXCR4 expression	3.35	1.90-5.92	<0.001
	N classification	1.88	1.42-2.49	<0.001
	Gender	0.49	0.24-0.98	0.046
**LRRFS**	UICC stage	1.76	1.22-2.54	0.002

*CI* confidence interval, *DMFS* distant metastasis-free survival, *HR* hazard ratio, *LRRFS* locoregional relapse-free survival, *OS* overall survival, *PFS* progression-free survival.

**Table 5:** Summary of the multivariate analysis of prognostic factors in nasopharyngeal carcinoma (Including ETAR and CXCR4)

**Clinical endpoint**	**Prognosticator**	**HR**	**95% CI**	***P *value**
**OS**	CXCR4 Expression	2.78	1.78-4.35	<0.001
	UICC Stage	1.51	1.15-1.98	0.003
	N classification	1.47	1.16-1.88	0.001
	Gender	0.48	0.29-0.80	0.004
	Age	1.65	1.07-2.56	0.024
**PFS**	CXCR4 Expression	2.30	1.42-3.72	<0.001
	ETAR Expression	2.12	1.03-4.36	0.04
	N classification	1.66	1.31-2.08	<0.001
	Gender	0.38	0.21-0.72	0.003
**DMFS**	CXCR4 expression	2.52	1.42-4.46	0.001
	ETAR expression	3.46	1.22-9.84	0.02
	N classification	1.96	1.48-2.59	<0.001
**LRRFS**	UICC stage	1.76	1.22-2.54	0.002

*CI* confidence interval, *DMFS* distant metastasis-free survival, *HR* hazard ratio, *LRRFS* locoregional relapse-free survival, *OS* overall survival, *PFS* progression-free survival.

### ET-1 induces CXCR4 mRNA and protein expression in 6-10B NPC cells

**Figure 2:**
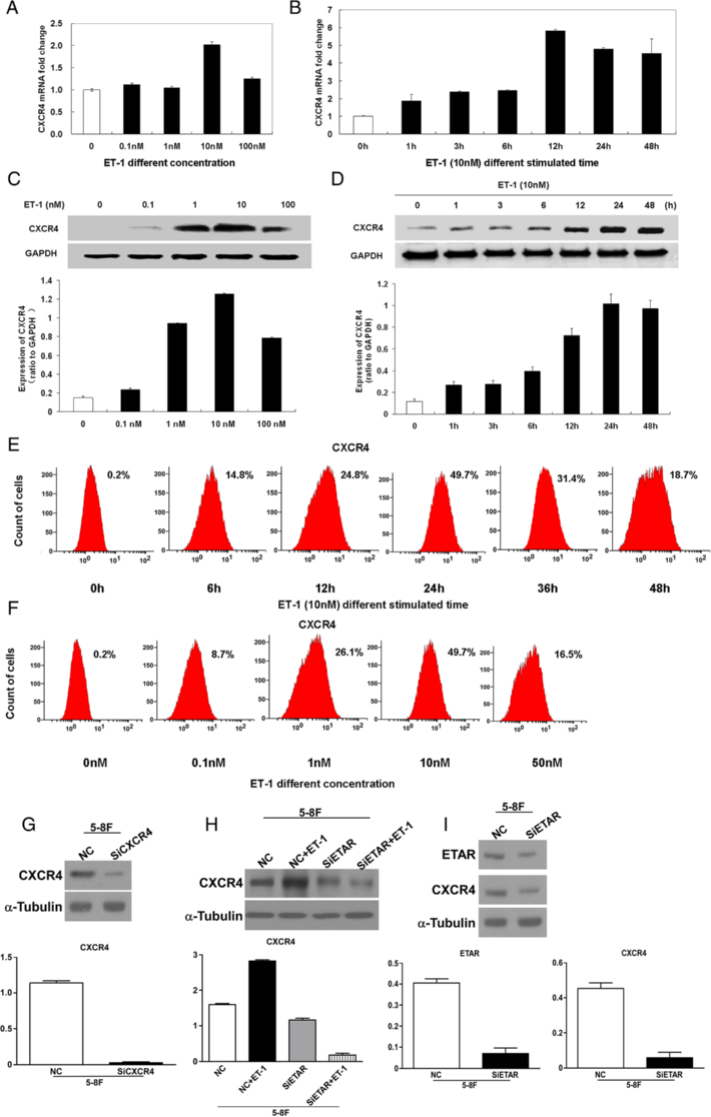
**ET-1 induces CXCR4 mRNA and protein expression in 6-10B nasopharyngeal carcinoma cells in a time- and concentration-dependent fashion (A-F).** Initially, 6-10B cells were serum-starved for 24 hours and then stimulated with increasing concentrations (0, 0.1, 1, 10, and 100 nM) of ET-1 for 24 hours or with 10 nM ET-1 for the indicated time. Total RNA was extracted and analyzed using real-time PCR for CXCR4 mRNA expression. Primers for GAPDH mRNA were used as a loading control **(A, B)**. Whole-cell lysates were prepared, and CXCR4 levels were analyzed by western blotting **(C, D)** and flow cytometry **(E, F)**. As an internal control, the membranes were reprobed with a specific anti-GAPDH antibody. CXCR4 induction was maximal in response to 10 nM ET-1 following a 24-hour exposure. Higher concentrations of ET-1 did not induce further CXCR4 expression. CXCR4 expression increased within 1 hour and remained high at 48 hours. siETAR reduced ETAR and CXCR4 protein expression and attenuated the ET-1-induced stimulation of CXCR4 expression in 5-8F cells **(G-I)**. siRNA transfection was performed per the protocol. Thirty-six hours after transfection, the cells were harvested, whole-cell lysates were prepared, and the CXCR4 and/or ETAR levels were analyzed by western blotting. α-tubulin was used as internal control.NC is a negative control with scrambled siRNA. Representative results from one of three independent experiments are shown.

We also investigated whether ETAR activation could increase CXCR4 expression in human NPC cells using real-time PCR for CXCR4 mRNA expression and western blotting and flow cytometric analysis for CXCR4 protein expression. The results showed that ET-1 induced CXCR4 mRNA and protein expression in 6-10B cells in a time- and concentration-dependent manner (Figure 2A-F).

### siETAR reduces ETAR and CXCR4 protein expression and attenuates ET-1 stimulation of CXCR4 expression in 5-8F cells

The knockdown of ETAR protein expression by siETAR reduced the expression of both ETAR and CXCR4 proteins, and ET-1 could not increase CXCR4 expression after ETAR knockdown in 5-8F cells (Figure 2G-I).

### ET-1 in combination with SDF-1α promotes 6-10B and 5-8F NPC cell migration

**Figure 3:**
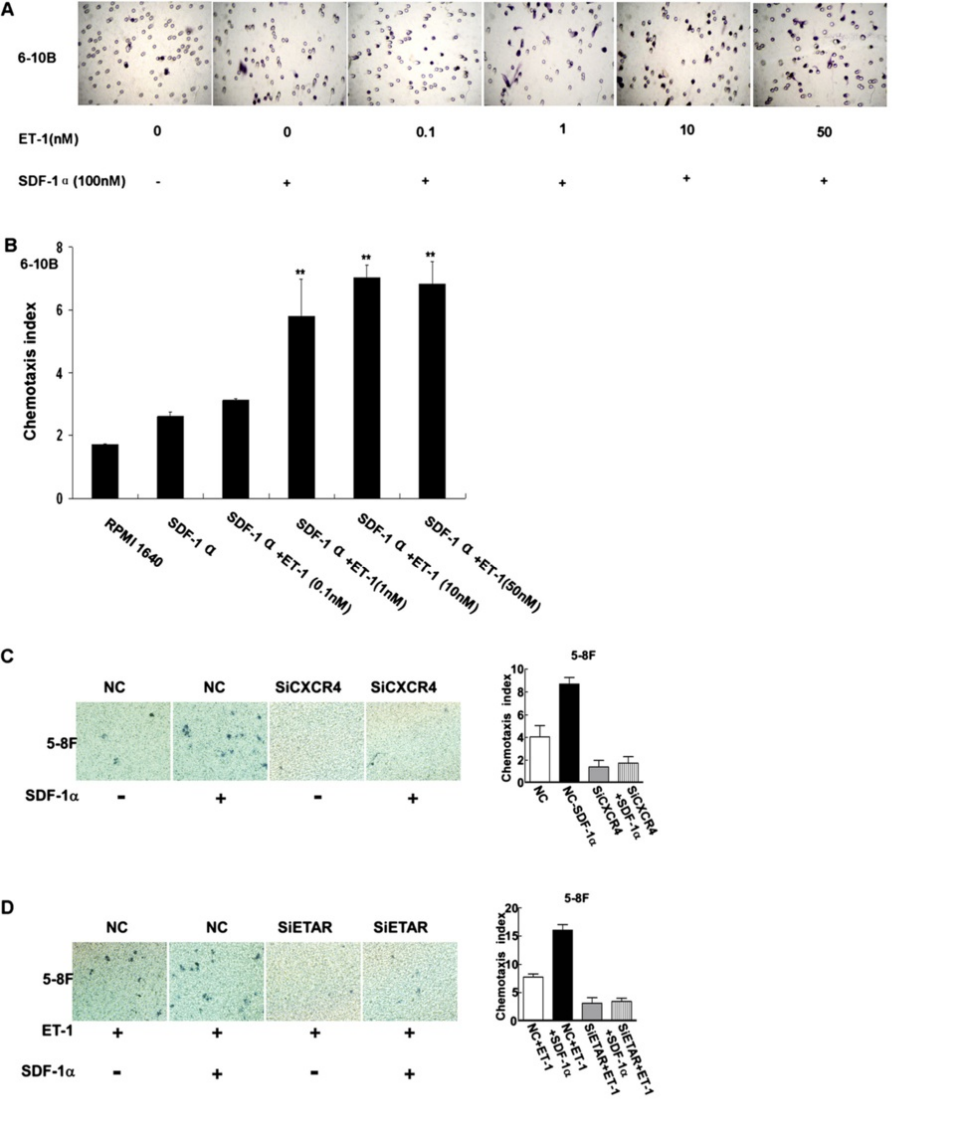
**ET-1 pretreatment augments the chemotactic activity of SDF-1**α **in 6-10B and 5-8F nasopharyngeal carcinoma cells. A-B**. The migration of 6-10B cells in response to SDF-1α was measured using chemotaxis chambers. Initially, 6-10B cells were serum-starved and then stimulated with increasing concentrations (0, 0.1, 1, 10, and 50 nM) of ET-1 for 12 hours, along with SDF-1α (100 nM), in the lower chamber. ET-1 stimulation together with SDF-1α in the lower chamber had an additive effect and produced a level of migration that was significantly higher than the level induced by SDF-1α alone. **C-D**. After transfection with siETAR and siCXCR4, ET-1 and/or SDF-1α no longer augmented chemotaxis in 5-8F cells. All panels show the mean ± SE of the migration index from at least three separate experiments, which were each performed in triplicate (Student’s unpaired t test; **P < 0.001 compared to SDF-1α alone).

A previous study showed that non-metastatic 6-10B NPC cells do not migrate in response to SDF-1α, despite the expression of CXCR4 by these cells [29]. Thus, the effect of ET-1 on 6-10B cell migration was examined using a Transwell assay. The results showed that 6-10B cell migration was stimulated by ET-1 (0.1-100 nM) in the presence of SDF-1α in a concentration-dependent manner. However, no migration was observed when the cells were treated in the absence of SDF-1α or with SDF-1α alone (Figure 3). Therefore, ET-1 upregulated the expression of functional CXCR4 and promoted the migratory ability of the 6-10B cells. In contrast, ET-1 no longer augmented CXCR4 expression in the 5-8F cells after ETAR knockdown, and a chemotaxis assay showed that ET-1 could not stimulate 5-8F cell migration, even with the application of SDF-1α.

### ET-1-induced CXCR4 expression in NPC cells is mainly mediated through ETAR

**Figure 4:**
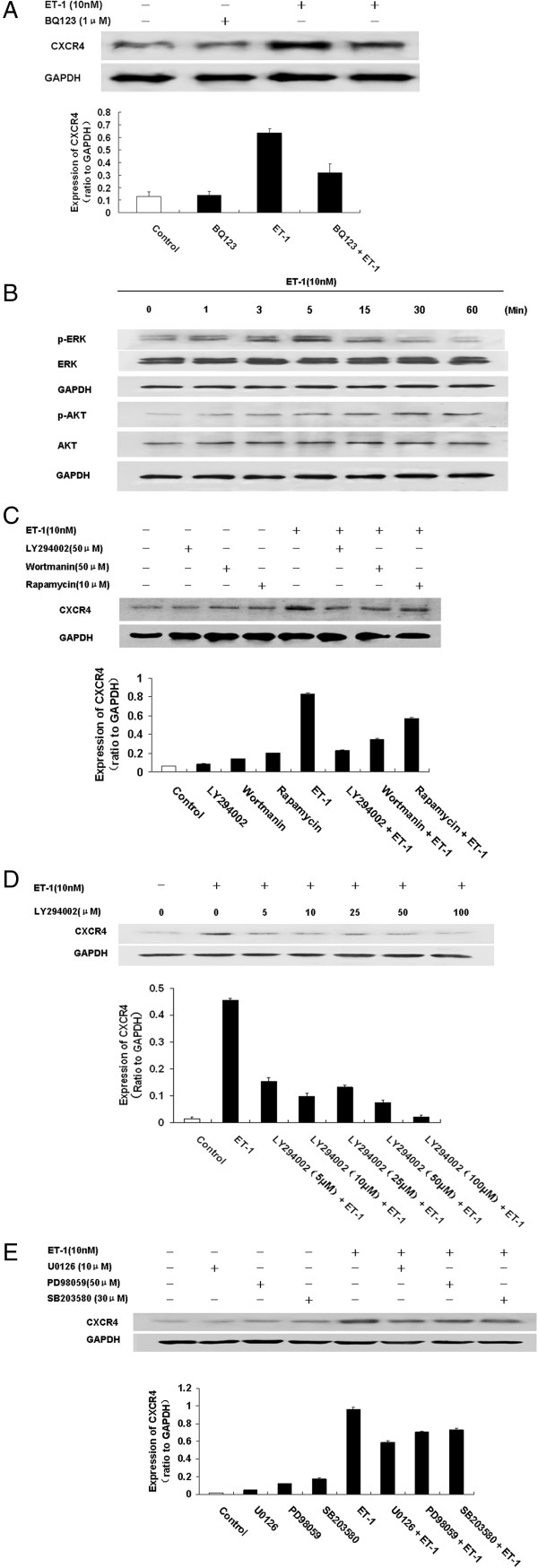
**The mechanism of ET-1-induced expression of CXCR4 in 6-10B nasopharyngeal carcinoma cells. A**. The ET-1-induced expression of CXCR4 in 6-10B nasopharyngeal carcinoma cells is mainly mediated through ETAR. Initially, 6-10B cells were serum-starved for 24 hours and pretreated with a selective ETAR antagonist, BQ123, for 2 hours. The cells were then either left unstimulated or stimulated with 10 nM ET-1 for 24 hours. The CXCR4 and GAPDH levels of whole-cell extracts were determined by western blotting. **B-E**. The ET-1-induced stimulation of CXCR4 expression was activated via the PI3K/AKT/mTOR or MAPK1/ERK1/2 signaling pathway in 6-10B nasopharyngeal carcinoma cells. **B**. Initially, 6-10B cells were serum-starved and treated with 10 nM ET-1 for various times, as indicated. Equal volumes of whole-cell lysate were analyzed by western blotting for AKT (phosphorylated on serine 473; P-AKT), total AKT, ERK1/2 (phosphorylated at threonine 202 and tyrosine 204; P-ERK1/2), total ERK1/2, and anti-GAPDH (loading control). **C**. Initially, 6–10 B cells were serum-starved and pretreated with LY-294002 (50 μM), wortmannin (10 μM), or rapamycin (50 μM) for 2 hours and then incubated in the presence of ET-1 (10 nM) for 24 hours. The CXCR4 levels were then analyzed by western blotting. **D**. The dose-dependent effects of LY-294002 on CXCR4 expression were evaluated in the presence of ET-1 (10 nM) for 24 hours. **E**. Initially, 6–10 B cells were serum-starved and pretreated with U0126 (10 μM), PD-98059 (50 μM), or SB203580 (30 μM) for 2 hours and then incubated in the presence of ET-1 (10 nM) for 24 hours. The CXCR4 levels were then analyzed by western blotting. ET-1 stimulation activated the AKT and MAPK signaling pathways, whereas blocking the PI3K/AKT or MAPK signal prevented ET-1-induced CXCR4 expression. Representative results from one of three independent experiments are shown.

In bladder cancer, ET-1 affects cell migration and invasion through ETAR. Accordingly, ETAR inhibitors have been suggested as potential therapeutic agents in advanced primary or metastatic bladder disease [34]. In the present study, we clarified the mediator responsible for ET-1-induced CXCR4 expression in NPC cells. ET-1 upregulated CXCR4 expression in the 5-8F cells, but CXCR4 expression was downregulated after ETAR was knocked down, and ET-1 could not stimulate CXCR4 expression after siETAR treatment (Figure 2I). Pretreatment of the 6-10B cells for 2 hours with the ETAR antagonist BQ123 (1 μM) markedly inhibited the expression of CXCR4 protein induced by ET-1 (Figure 4A). These results indicated that ETAR was the mediator of ET-1-induced CXCR4 expression.

### ET-1 upregulates the expression of CXCR4 via the PI3K/AKT and MAPK/ERK1/2 pathways

To explore the signaling mechanism responsible for ET-1-upregulated CXCR4 expression, immunoblotting was used to observe alterations in the levels of phosphorylated ERK and AKT after the pretreatment of 6-10B cells with 10 nM ET-1. ERK phosphorylation began at 1 minute after ET-1 treatment and reached its maximum in 5 minutes, though the level was significantly reduced 30 minutes later (*P* < 0.05). AKT phosphorylation began at 1 minute after ET-1 treatment and reached its maximum in 30 minutes; the level was significantly reduced after 60 minutes (*P* < 0.05). These results suggested that the ET-1-induced upregulation of CXCR4 expression in the NPC cell line 6-10B might be mediated by the phosphorylation of ERK and AKT (Figure 4B). Interestingly, total ERK did not change significantly during the progression, whereas total AKT slightly increased.

To further investigate whether the ET-1-induced upregulation of CXCR4 occurred through the PI3K/mTOR signaling pathway, 6-10B cells were incubated in the presence of the PI3K inhibitors LY294002 and wortmannin and the mTOR inhibitor rapamycin prior to the administration of ET-1. LY294002 (0, 5, 10, 25, 50, and 100 μM), wortmannin (10 μM), or rapamycin (50 μM) were added to pretreat the cells for 2 hours prior to the addition of 10 nM ET-1 for 24 hours. The results show that CXCR4 expression was significantly enhanced after 24 hours when ET-1 was added in the absence of these inhibitors; however, the CXCR4 protein level was decreased when ET-1 was added to the cells after pretreatment with an inhibitor. Specifically, LY294002 administration resulted in a dose-dependent decrease in ET-1-induced CXCR4 expression. Thus, ET-1 promoted the expression of CXCR4, whereas the PI3K inhibitors LY294002 and wortmannin and the mTOR inhibitor rapamycin inhibited the upregulation of CXCR4 by ET-1 (*P* < 0.05) (Figure 4C). Specifically, administration of the PI3K inhibitor LY294002 resulted in a dose-dependent decrease in ET-1-induced CXCR4 expression (Figure 4D).

We also examined the role of the MAPK/ERK1/2 signaling pathway in ET-1-induced CXCR4 upregulation. The cells were pretreated with the MEK inhibitor U0126 (10 μM), the ERK1/ 2 inhibitor PD98059 (50 μM), or the P38MAPK inhibitor SB203580 (30 μM) for 1 hour prior to the administration of 10 nM ET-1 for 24 hours. The results show that ET-1 treatment in the absence of inhibitor resulted in the upregulation of CXCR4 expression. However, ET-1 treatment following pretreatment of the cells with one of these inhibitors resulted in a mild decrease in CXCR4 expression (*P* < 0.05) (Figure 4E). Based on these results, it appears that the MAPK/ERK1/2 signaling pathway is a second pathway involved in ET-1-induced CXCR4 upregulation in 6-10B cells.

Taken together, these data suggest that ET-1 activates the PI3K/AKT/mTOR and MAPK/ERK1/2 signaling pathways via ETAR and then upregulates CXCR4 expression in 6-10B NPC cells.

## Discussion

Distant metastases are the most frequent cause of death in patients with NPC. In our previous study [8], we demonstrated that NPC patients had a high plasma level of ET-1, which correlated positively with metastasis and was an independent prognostic factor in these patients. ABT-627, an antagonist of ETAR, can significantly inhibit the growth of NPC xenografts in nude mice, reduce metastatic lesions in the lung, and enhance the sensitivity of the tumors to chemotherapy [9]. The present study showed that ETAR overexpression was associated with distant metastasis in NPC patients, consistent with the results of others [27,28]. The ET-1/ETAR pathway regulates tumor invasion and metastasis in many processes, including adherence, mobility, the epithelial-mesenchymal transition (EMT), the secretion of degradation enzymes, angiogenesis, bone deposition in bone metastasis, and the formation of lymph vessels [35-37].

The present study showed that CXCR4 overexpression was associated with distant metastasis in NPC patients. In 2005, Hu et al. [28] were the first to demonstrate that the CXCL12/CXCR4 axis plays a pivotal role in NPC spread and specific organ metastasis, providing an important clue regarding the mechanisms involved in NPC metastasis. Indeed, CXCR4 has been reported to be a prognostic marker in various types of cancer, such as acute myelogenous leukemia [38] and breast carcinoma [39]. The specific expression of chemokines and their receptors is an important process in malignant tumor cells that are prone to metastasize to remote organs. Balkwill [40] reviewed studies demonstrating that malignant cells from different types of cancer express CXCR4 and interact with its ligand, SDF-1, indicating the critical role that the SDF-1/CXCR4 pathway plays in tumor metastasis. SDF-1 (also known as CXCL12) is a chemotactic protein secreted by bone marrow stromal, mesothelial, and epithelial cells. CXCR4 is the only known receptor for SDF-1 and has a high affinity for this chemokine. The binding of CXCL12 to CXCR4 induces intracellular signaling through several divergent pathways, initiating signals related to chemotaxis, cell survival and/or proliferation, increased intracellular calcium, and gene transcription. The CXCL12/CXCR4 axis is involved in tumor progression, angiogenesis, metastasis, and survival [41], and promising results in preclinical tumor models indicate that CXCR4 antagonists may have antitumor activity in patients with various malignancies [42]. Smith et al. [43] found that inhibiting CXCR4 with RNAi or the specific antagonist AMD3100 substantially delayed the growth of 4 T1 breast cancer cells in the lungs of BALB/c mice. These results extend the potential therapeutic applications of CXCR4 inhibitors to the treatment of both primary and metastatic breast cancer.

In the present study, we evaluated the expression of ETAR and CXCR4 in NPC using immunohistochemistry. To the best of our knowledge, we are the first to show that ETAR expression is closely associated with CXCR4 expression in patients with NPC. As both ETAR expression and strong CXCR4 expression are associated with unfavorable PFS and DMFS, it is interesting to evaluate the relationship between ETAR and CXCR4 expression. We speculated that there may be crosstalk between the ET-1/ETAR and SDF-1α/CXCR4 pathways, and our study indicated that the expression levels of ETAR and CXCR4 were positively correlated. In the 48 NPC cases positive for the expression of CXCR4, 95.8% also exhibited ETAR expression, and our experimental study also showed that ETAR activation increases functional CXCR4 expression in 6-10B and 5-8F NPC cells. Both the 5-8F and 6-10B cell lines are sub-clones of the NPC cell line SUNE1 [33]: the 5-8F cell line has the potential for high tumorigenesis and high metastasis, whereas the 6-10B cell line has the potential for tumorigenesis but cannot metastasize. Qiu et al. [44] found that the expression level of CXCR4 is higher in 5-8F than in 6-10B cells, and another study has shown that the 6-10B cell line expresses CXCR4 but that the receptor is inactivated [28]. It was also found that the ability of 5-8F cells to proliferate and migrate increased after SDF-1α stimulation, though no significant changes occurred in the 6-10B cell line [28]. In the present study, we found that pretreatment with ET-1 augments the chemotactic activity of SDF-1α in the 6-10B NPC cell line via the upregulation of the expression of functional CXCR4. Our results suggested that the ET-1/ETAR pathway may play an important role in CXCR4 expression in NPC. Our results also revealed an association between ETAR and CXCR4 expression, though the multivariate analyses showed that the two expression levels are independent of each other. However, it should be noted that we applied multivariate analyses to prognostic research and that the factors that have an effect on prognosis are very complicated. For example, ET-1/ETAR may also promote cancer metastasis by regulating VEGF [45,46], matrix metalloproteinase [47,48], hypoxia-inducible factor-1alpha (HIF-1α) [49], and the epithelial-to-mesenchymal transition [50]. Thus, the association between ETAR and CXCR4 that we revealed based on clinical data only shows that the receptors are correlated in quantity.

The present study showed that ET-1 induced CXCR4 expression by activating the PI3K/AKT/mTOR and/or MAPK/ERK1/2 signaling pathways. Our study also showed that ET-1-induced CXCR4 expression could be inhibited by an ETAR antagonist or an inhibitor of PI3K/AKT/mTOR or MAPK/ERK1/2. In fact, CXCR4 can be regulated by many pathways. A study by Segawa et al. [51] demonstrated that high levels of CXCR4 and VEGF correlate with a poor prognosis in NPC patients, and Bachelder et al. [52] demonstrated that VEGF promotes breast cancer tumor cell invasion via the upregulation of CXCR4 expression.

Many studies have revealed a close relationship between CXCR4 and the PI3K/Akt/mTOR or MEK/ERK pathway. Kukreja et al. [53] reported that CXCL12 upregulates CXCR4 via activation of the MEK/ERK and NF-kB pathways in prostate cancer cells. In hepatocyte growth factor (HGF)-treated MCF-7 cells, Maroni et al. [54] demonstrated that the DNA binding of Ets1, activated by the MAPK/ERK1/2 transduction pathway, and the DNA binding of NF-kB played a critical role in CXCR4 transcription and protein induction and enhanced the invasion and migration ability of the breast cancer cells. Phillips et al. [55] demonstrated that EGF and hypoxia upregulate CXCR4 via the PI3K/AKT/mTOR pathway and the activation of HIF-1α in NSCLC. Lastly, Yu et al. [56] demonstrated that CXCR4 induces MMP-9 and MMP-13 expression and promotes the invasion ability of oral squamous carcinoma via the ERK pathway.

Collectively, our observations revealed that ETAR and CXCR4 are important molecules involved in the spread and progression of NPC cells. ETAR activation promoted NPC migration and was associated with a poor prognosis via a mechanism that involves, at least in part, increasing functional CXCR4 expression. Drugs targeting the endothelin axis, such as the potent ETAR antagonist atrasentan (ABT-627), have been studied in large clinical trials and appear to have an impact on disease progression and morbidity [57]. Several inhibitors/antagonists (e.g., T140, AMD3100) have recently been generated and theoretically may block direct interactions between CXCR4 and CXCL12. Because of the critical role that the CXCL12/CXCR4 axis plays in HIV infection and cancer metastasis, it has served as an important target in the development of antitumoral and anti-HIV-1 therapies [58]. Targeting ETAR and CXCR4 at the same time may be a potential therapy for preventing the metastasis of NPC. Hence, our findings may be useful in the future development of novel strategies for targeting NPC tumor metastasis.

## Conclusion

Our study revealed that elevated ETAR and CXCR4 expression is correlated with distant metastasis and poor survival in NPC patients and can serve as an independent prognostic factor in NPC patients. Thus, ETAR and CXCR4 may be useful predictors of NPC prognosis. ET-1 promoted NPC cell motility by elevating the level of functional CXCR4 through the activation of the PI3K/AKT/mTOR and/or MAPK/ERK1/2 signaling pathways. ET-1 may play an important role in regulating CXCR4 expression in NPC cells; however, the mechanisms underlying how ET-1 regulates CXCR4 are complex and warrant further study.

## Abbreviations

NPC: Nasopharyngeal carcinoma; ETAR: Endothelin A receptor; CXCR4: Chemokine receptor 4; SDF-1: Stromal-derived factor-1; ET-1: Endothelin-1; VEGF: Vascular endothelial growth factor; MAPKs: Mitogen-activated protein kinases.

## Competing interests

The authors declare that they have no competing interests.

## Authors’ contributions

DHL, QYC, and HQM conceived the study design, collected the clinical data from patients, performed and coordinated the immunohistochemical examinations of tumor specimens, and drafted the manuscript. HL and LHX performed the IHC staining and biochemical experiments. HZZ conducted the immunohistochemistry analysis. LZ, LQT, and PYH participated in collecting the clinical data from patients. HYM and XG participated in the interpretation of the data. DHL performed the statistical analysis of the data. All authors have read and approved the final version of the manuscript.
